# Leipzig - Individual Placement and Support for people with mental illnesses (LIPSY): study protocol of a randomized controlled trial

**DOI:** 10.1186/s12888-021-03416-7

**Published:** 2021-08-19

**Authors:** Felix S. Hussenoeder, Maria Koschig, Ines Conrad, Uta Gühne, Alexander Pabst, Sophie-Elisabeth Kühne, Mathias Alberti, Katarina Stengler, Steffi G. Riedel-Heller

**Affiliations:** 1 Institute of Social Medicine, Occupational Health and Public Health, University of Leipzig, Ph.-Rosenthal-Str. 55, 04103 Leipzig, Germany; 2 Helios Park-Klinikum - Clinic for Psychiatry, Psychosomatics and Psychotherapy, Morawitzstr. 2, 04289 Leipzig, Germany

**Keywords:** Mental health, Severe mental illness, Work, IPS, RCT, Labor market integration, Intervention, Supported employment, Vocational rehabilitation

## Abstract

**Background:**

Individuals receiving means-tested benefits are at a higher risk of being diagnosed with a psychiatric illness compared to those who are employed, and the rate of those working in the first labor market is low. The intervention (Individual Placement and Support, IPS) aims at maintaining or regaining working ability and at facilitating reintegration into the (first) labor market following a “first place, then train”-approach. The objective of the study is to conduct the first RCT in Germany that addresses a broad group of long-term unemployed individuals with severe mental illnesses that receive means-tested benefits, and to test the effectiveness of the IPS intervention.

**Methods:**

In this randomized controlled trial, about 120 eligible participants aged between 18 years and local retirement age will be randomly allocated to an intervention group (IG) or to an active control group (CG) using a parallel arm design. The IG will receive IPS + high quality treatment as usual (TAU), the active CG will receive TAU + a booklet on integration measures. A block-randomization algorithm with a targeted assignment ratio of 1:1 for participants in IG and active CG will be used, stratified by sex and three age groups. Assessments will take place before the intervention at baseline (t0), and 6 (t1), 12 (t2), and 18 (t3) months later. Primary outcome will be the proportion of participants having worked at least 1 day in competitive employment since baseline, as assessed at t3. Secondary outcomes will be related to employment/ vocation and mental health. In addition, there will be a process evaluation. Treatment effects on outcomes will be tested using appropriate panel-data regression models, and acceptability, uptake and adherence will be evaluated using descriptive statistics and appropriate inference testing.

**Discussion:**

The results of this trial are expected to generate a better understanding of the efficiency, feasibility, acceptance, and relevance of the IPS intervention in a German setting. They could be a first step towards the implementation of the method and towards improving the situation of long-term unemployed individuals with severe mental health problems.

**Trial registration:**

German Clinical Trials Register (DRKS00023245), registered on 22.02.2021.

## Background

Work is an essential part of daily life, and it is especially important for individuals with mental health problems [1]. It does not only provide a certain level of financial security and time structure, but also facilitates social interactions and mental health. A review of meta-studies by Paul et al. [2] shows the connection between mental health and unemployment: 34% of unemployed individuals were affected by mental health problems, double the number of their employed counterparts. Already in 2006, cross-sectional studies demonstrated a negative connection between unemployment and wellbeing in all six dimensions, i.e., unspecific symptoms, depression, anxiety, psychosomatic symptoms, subjective wellbeing, and self-esteem [3].

For a long time, there was no information available on how many of those receiving means-tested benefits in Germany also suffered from mental illnesses. In 2013, a study [4] provided this information using data from six different health insurance companies. It shows that 37% of insured individuals that received means-tested benefits were diagnosed with at least one psychiatric disorder, and that psychiatric diagnoses were on the rise. More than a fifth received a diagnosis in the area of “neurotic/ stress/ somatoform disorder”, and about every sixth was diagnosed with an affective or an addictive disorder. Schizophrenia, personality disorders, and behavioral disorders were much rarer. In 2009, about one out of seven individuals receiving means-tested benefits experienced depressive episodes, followed by somatoform disorders (1 out of 10). In addition, anxiety disorders, reactions towards heavy strain, and adjustment disorders played a role.

While there is a broad range of vocational (re) integration measures for people with mental illnesses in Germany, the rate of those working in the first labor market is low [5]. At the same time, the number of individuals with mental illnesses working in workshops for persons with disabilities is growing steadily, currently reaching 21% [6]. Many are also unemployed and receive benefits [4].

There seems to be much room for improvement regarding the cooperation between relevant stakeholders in healthcare, rehabilitation, and at jobcenters to support unemployed persons with mental illnesses [5]. In addition, new approaches are needed in the German rehabilitation system [7]. Until now, vocational integration efforts are selective and mainly tied to specific institutions with high entry barriers. Most of the time they follow an approach best described as “first train then place”, i.e., a preparatory training in a protected environment followed by the integration into the first labor market. Internationally, successful measures use individualized approaches with job coach support known as individual placement and support (IPS), and with the goal to quickly place an individual in the first labor market, i.e., „first place then train “ [8]. These approaches are also recommended by the S3 guidelines „Psychosocial therapies in severe mental illness [9]. Meta-analyses and reviews show that IPS is superior to traditional vocational rehabilitation in terms of achieving competitive employment and with regard to multiple other vocational outcomes like job tenure and total income [10, 11]. This holds also true in specific groups like people diagnosed with a psychotic illness [12], patients with offending histories [13], or young adults [14]. All of the previous trials were conducted outside of Germany, e.g. more recently in Norway [15] and the UK [16]. So far, only the multi-centric EQOLISE trial tested IPS in different European counties including a German study center [12]. Although the trial was in favor of IPS, results for the German study center were not significant. However, there was a tendency for participants in the IPS-group, as compared to those in the control group that received an alternative rehabilitation measure, to gain employment more often, work more hours and days, and keep employment for a longer duration [17]. Although the trial was powered for the multi-centric overall result, this discrepancy provoked national discussion [18].

We therefore want to fill a gap in research by conducting the first RCT in Germany that addresses a broad group of individuals with severe mental illnesses.

### Objectives

The objective of the LIPSY trial is to test the effectiveness of an IPS intervention in a German context, and with long-term unemployed individuals with severe mental illnesses that receive means-tested benefits. The intervention aims at maintaining or regaining their working ability and at facilitating their reintegration into the first labor market. We will test the hypothesis that IPS is superior to the treatment of the active control group (a booklet on integration measures). Specifically, we assume that participants will be significantly more likely to have worked in competitive employment for at least 1 day at t3 (18 months after intervention, primary outcome) if they received IPS (IG) as compared to not receiving IPS (CG). In addition, we will analyses the effects of the intervention on a wide range of vocational and health-related outcomes. Therefore, the present study closes a research gap in Germany, and if proven effective may further close a supply gap for reintegration of individuals with severe mental health problems.

## Methods

### Design and setting

The study will implement a randomized controlled trial (RCT) using a parallel arm design with allocation of participants to either an intervention group (IG) or an active control group (CG). Both groups will receive high quality treatment as usual as a standard treatment at a clinic for psychiatry, psychosomatics and psychotherapy (TAU), with participants in the IG additionally receiving IPS, and participants in the active CG additionally receiving a booklet on current measures for vocational rehabilitation. The study will take place in a clinical setting in Leipzig, a major city in Eastern Germany. The trial was registered at the German Clinical Trials Register on 22.02.2021 (DRKS00023245).

### Inclusion and exclusion criteria

Participants will be included based on the following inclusion criteria: (a) receiving means-tested benefits (German: ALG 2); (b) severe mental illness (GAF ≤ 50) as a main diagnosis (ICD-10) excluding acute intoxication and delirium; (c) age 18+ years up to local retirement age; (d) an expressed moderate to strong desire/ wish to work; (e) being willing and capable of giving informed consent; and (f) receiving treatment at an outpatient psychiatric clinic. Participants will be excluded if they do not fulfill the inclusion criteria.

### Interventions

Participants from both groups will receive high-quality TAU, i.e. the standard treatment according to their needs by a multiprofessional team at the outpatient psychiatric clinic. This can include medication, psychotherapy and a wide set of optional psychosocial therapies, like social skills training. In addition, participants in the IG will receive IPS, and participants in the active CG will receive a booklet that gives an overview on measures for vocational rehabilitation. There are no restrictions with regard to concomitant care.

#### Intervention group

IPS coaching is focused on the individual needs and goals of participants, and it covers all phases of vocational orientation and goal development, job application, beginning to work, workplace adaption, job retention, job loss, and (re)orientation. If they want to, participants can involve a related person and/or their employers in the coaching process, and coaches can meet participants outside the hospital. In addition, coaches build up contacts to potential employers in general. There will be a basic documentation of meetings, talks and communication between coaches and participants including key issues and agreements. All coaches are part of the clinic team, received an online training with certification (https://ipsworks.org/index.php/training-courses/), and they work manual-based [19]. Coaches cooperate closely with physicians, social workers, and therapists.

The coaching starts with a planning phase in which the needs of participants are assessed in detail and personal goals are defined. Specific questionnaires can be used to explore potential goals, strengths, and experiences of participants; they may also address cognitive abilities, social competence, qualifications, and daily activities, depending on the needs of the participants. Besides starting a job or changing occupations the exploration may also yield other goals like the acquisition of new qualifications, the beginning of studies, or an apprenticeship. Goals will be written down in an objective agreement which includes statements on vocational preferences, companies that will be addressed, and participant’s needs.

In the intervention phase, goal achievement will be addressed, for example, employers could be contacted. It is important that the intervention phase starts early, best in the first 30 days after the beginning of the coaching process. Once the participant has managed to start work/ training/ studies (integration phase), the coaching will be adapted to this new situation. For example, job coaches can then give the following support: meeting with family members to explore job-related strengths and goals; meeting with job center staff to coordinate planning; job search and preparation of job interviews; time management; social skills training; communication with the employer, e.g., for conflict mediation or workplace adaption; workplace coaching; group sessions with other participants.

The productive contact with employers is a crucial success factor. Therefore, it will be discussed at an early stage if, and in which form, it could make sense for participants to reveal their illness to their potential employer. Participants’ preferences with regard to the disclosure of their mental health status by their coach to a potential employer will be documented and binding. If a participant decides to disclose their condition, the contact between coach and employer will be documented. The participant can withdraw their agreement at any time.

Participants will be coached steadily, if requested also after the project has ended. Continued support via the hospital’s own social services is available. Due to the close contact between coach and participant, potential barriers can be identified at an early stage and individual solutions can be developed. Should participants miss appointments or not follow agreements, coaches will actively seek contact for 8 weeks after the last contact. Participants can withdraw from the program at any time. In addition, the IPS-process includes multiple opportunities for participants to give feedback and shape the process, which are expected to contribute to intervention adherence.

#### Active control group

Participants in the active CG will receive TAU and a booklet since a pure control group with no intervention would be neither feasible nor legally or ethically acceptable. The booklet gives an overview on current measures for vocational rehabilitation for individuals with mental illnesses.

### Assessments

We will collect data on sociodemographic variables and a wide set of characteristics related to employment situation, educational activities, additional work rehabilitation measures, health, disability, treatment, participation expectancy, and desire to work via a study-specific questionnaire. For example, items address citizenship, health status (e.g., need for care), employment history (e.g., “How many years did you work in competitive employment?”), psychotropic medication, and psychotherapy.

In addition, psychologists from the clinic will perform a clinical diagnosis of participants. They will forward information on the diagnosis (ICD-10) and the global level of functioning (GAF) to the ISAP research team using the project-ID of participants. The presence of a severe mental illness (GAF ≤ 50) as a main diagnosis, excluding acute intoxication and delirium, is also a key inclusion criterion.

Perceived social support will be assessed via the short form of the *Lubben Social Network Scale (LSNS-6)*, containing six items that can be rated on 6-point Likert scales [20]. Attitudes toward recovery from psychiatric disorders will be assessed via the Recovery Attitudes Questionnaire (RAQ-7; seven items [21];). Participants will be asked for their subjective prognosis of gainful employment via the *“Subjektive Prognose der Erwerbstätigkeit”-Skala* (*SPE*, [22]) that contains three items which can be answered on a 5-point Likert scale. Loneliness will be measured with the 3-item SOEP-version of the *UCLA loneliness scale* (5-point scale [23, 24];). Optimism and pessimism will be measured with the revised version of the *Life-Orientation-Test (LOT-R)* via six items (5-point scales, filler items excluded [25, 26];). We will assess health care consumption in eight different areas, e.g., living situation, hospital stays, and medication, via a shortened version of the „Fragebogen zur Inanspruchnahme medizinischer und nicht medizinischer Versorgungsleistungen bei psychischen Erkrankungen “(FIMPsy, [27]). The 36-item *WHO Disability Assessment Schedule* (*WHODAS 2.0* [28];) will be used to measure general health and disability levels in six different domains (*cognition, mobility, self-care– hygiene, dressing, getting along, life activities, participation*) at t0, but we will remove four items related to work. At t3 the 12-item version of the instrument will be applied. In addition, we will use original items to measure substance use.

Cognitive performance will be assessed by trained staff at the clinic via the *SCIP-D* (*Screen for Cognitive Impairment in Psychiatry – German version* [29];), a brief screening tool to assess cognitive impairment in psychiatric patients in five areas (*immediate verbal learning, delayed verbal learning, working memory, verbal fluency, processing speed*).

### Primary outcome

Our primary outcome is the proportion of participants “having worked at least one day in competitive employment” (yes vs. no) during the entire observation period upon t3 (18 months after baseline). We define competitive employment as employment that any person can apply for regardless of disability status. Workers earn minimum wage or higher, and self-employment is also considered to be competitive employment (also see ipsworks.org). It will be assessed as self-reported information via questionnaire.

### Secondary outcomes

We will use items to assess vocational variables from t1 to t3, i.e., time until first competitive employment (days), job tenure (days), number of different employments, working time per week (hours), overall time in employment (days), reason for the termination of the first competitive employment (if applicable), current employment status and job position, current employment situation (e.g., permanent full time), and wage/ income.

The following instruments and items will be assessed at every time point, from t0 to t3. The general state of health will be measured with the *EuroQol* visual analogue scale (EQ-VAS) from the *EQ-5D* (e.g., [30]), a thermometer-like scale on which participants can rate their health from worst (=0) to best (=100) imaginable health. Physical and mental health will be assessed with the 12-item *Short-Form Health Survey (SF-12*, different scale formats [31];), and depressiveness with the *Patient Health Questionnaire-9 (PHQ9)*, which contains 9 symptom-oriented items that can be rated from 0 (= not at all) to 3 (= almost every day) [32–34]. In addition, we will use the *Mini-Symptom-Checklist* that includes three sub-scales with six items each (*Depression, Anxiety, Somatization*) and the 5-item *Aggression/Hostility*-subscale from the *Brief-Symptom-Checklist* which can be rated on 5-point Likert scales (*Mini-SCL, BSCL* [35, 36];). The 3-item *Alcohol Use Disorders Identification Test (AUDIT-C)* will be used to assess problematic alcohol consumption [37]. In order to measure participants self efficacy with regard to their return to work, we will use the 11-item *return-to-work self-efficacy scale* (*RTW-SE* [38, 39];).

The following instruments will be used at two time points, t0 and t3. The *Working Ability Index (WAI)* consists of seven dimensions that can be rated on different scales yielding end results between 7 (= critical working ability) and 49 (= very good working ability) points [40]. Since the *WAI* to a large extend addresses individuals that are working, we will utilize a reduced scale at t0. We will measure attitudes towards work (6 items) and working motivation/wish to change (2 items) with items from the “*Diagnostikinstrumente für Arbeitsmotivation*” (*DIAMO* [41];), and vocational self efficacy with the “*Skala zur Erfassung beruflicher Selbstwirksamkeitserwartungen*” (*BSW* [42];).

An overview of all variables, outcomes and other measures that will be implemented is shown in Table 1.

**Table 1:**
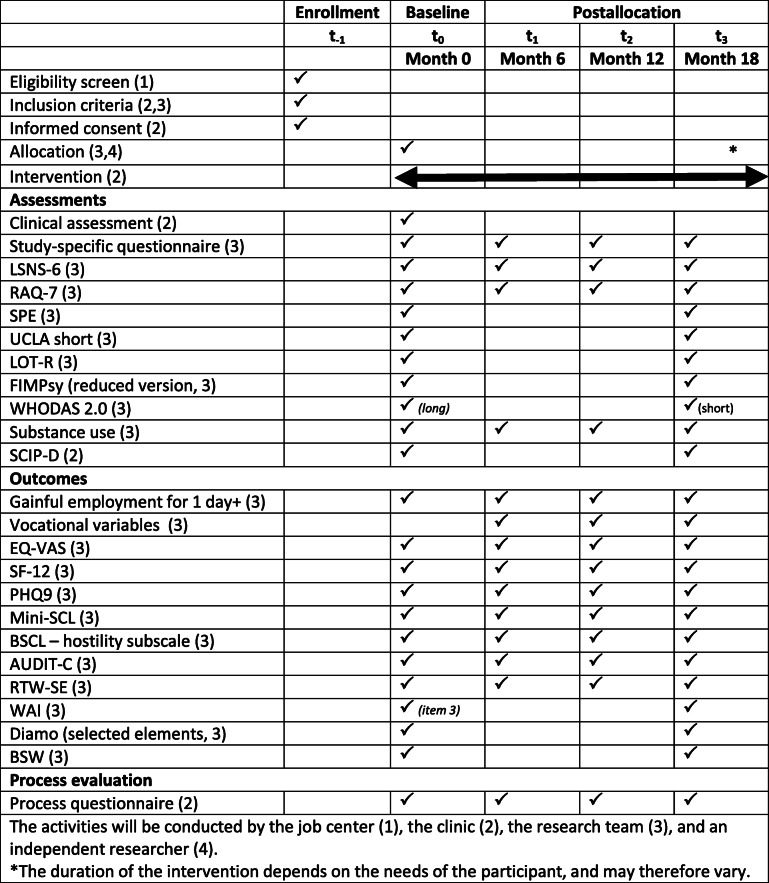
Overview of enrollment, assessment, and evaluation of the LIPSY-trial

### Participant timeline and recruitment

**Fig. 1:**
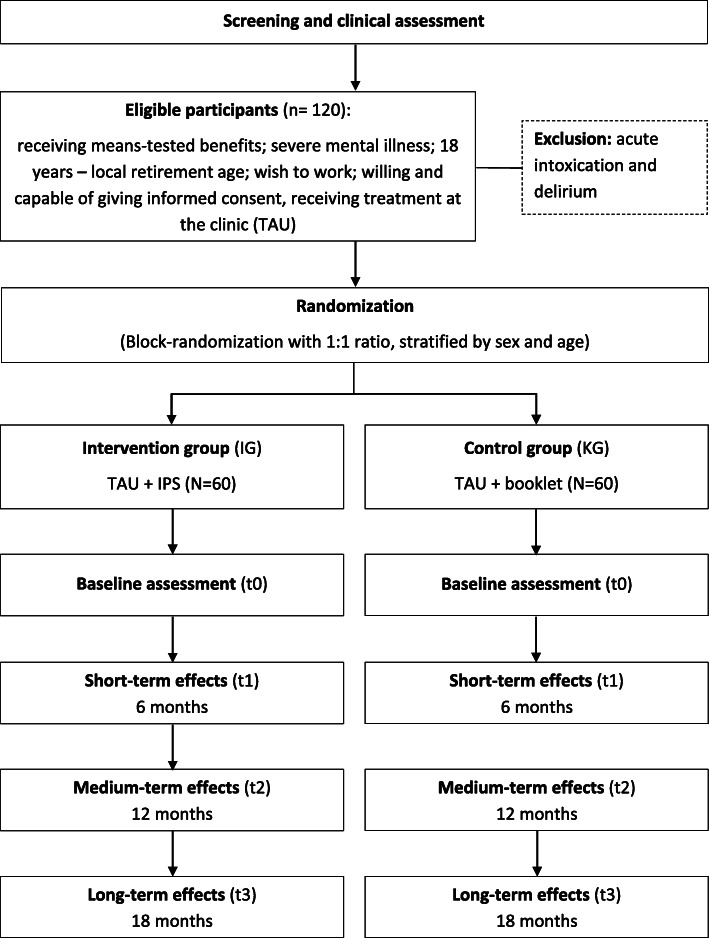
Flow of participants in the LIPSY-Trial (TAU = treatment-as-usual, IPS = Individual Placement and Support)

Potential participants will be recruited through the Jobcenter Leipzig in the months previous to the intervention, where they will receive a screening test that will be used to identify potential participants that receive means-tested benefits and suffer from a mental health problem. If tested positive in the screening, they will receive a detailed information sheet on the goals, nature, and implications of the trial as well as on data protection regulations and will be asked to sign a consent form. Potential study participants will be given sufficient time to consider their participation and to ask questions. The psychologists that are responsible for the first assessment in the trial are directly located in the job center to reduce the threshold for participation. Due to the sustained Covid-19 pandemic, we will also consider alternative ways of recruiting, e.g., via written invitations and directly at the clinic. We are planning to start the intervention with the first participants in April 2021 and to include the last participant in December 2022. Since IPS is highly individualized and need-oriented, there are no strict time schedules for the intervention, and IPS may be going on even after the project is finished. Enrolled participants will be assessed at baseline (t0) and after 6 (t1), 12 (t2), and 18 months (t3). We offer the possibility to partake in a project that is aimed at improving the situation of our target group and where participants receive support that can help them to better find work and improve their mental health which we expect to promote participant motivation and retention. Figure 1 shows the flow of participants in the LIPSY trial.

### Sample size

Sample size calculation is based on the primary outcome measure (competitive employment for at least 1 day). In the IPS-literature there is only one European Trial with data from a German study center [12]. The proportions of the EQOLISE-Trial were applied in the power calculation (IG: 0.55, CG: 0.28). To detect a between-group effect of ∆ = 0.27 in favor of IG at 18 months, considering a significance level of α = 0.05 (one-sided) and a statistical power of 1 - β = 0.80, we estimated a target sample of *n* = 41 participants per arm is needed. Anticipating a drop-out rate of 30% until 18 months after baseline assessment based on experiences with previous clinical research projects, the total sample size would have to comprise roughly 60 individuals per group.

### Assignment of interventions: allocation and blinding

Eligible persons who consented to participate will be randomly assigned to either IG or active CG using a computerized random number generator. A block-randomization algorithm with a targeted assignment ratio of 1:1 for participants in IG or active CG will be used, stratified by sex and three age groups: 18–35, 36–50, 51+. This facilitates balance between study arms in both sample size and basic demographic variables. Stratified randomization will be achieved by using a separate randomization procedure within each of the strata.

Blinded randomization will be done by an independent statistician, generating randomization block lists with respective statistics software. The randomization lists (one for each stratum) will be concealed to the study coordinator and recruiting staff members with regard to the lists’ strata identity. Employees at job center and clinic who recruit eligible participants and collect data, will remain blind to the group allocation. Participants of both IG and active CG cannot be blinded to the intervention they receive, because blinding is per se not possible when conducting an IPS intervention. However, the statistician analyzing the data and staff members evaluating the results will remain blind to the group allocation.

An independent researcher will generate the allocation sequence. Psychologists from the clinic will enroll participants and inform the researchers about the enrollment who will then initiate randomization and assign the participants to one of the groups.

### Data collection and management

Baseline assessments will be conducted using written questionnaires handed out to participants at t0. Follow-up assessments (t1 - t3) will be scheduled 6, 12, and 18 months after the baseline assessment, and participants will be invited to fill out the questionnaires in the presence of project staff, so that they can ask for support or clarification if they wish to. In addition, we plan to include register data from the job center on the current working status of participants at t3. An overview of all instruments utilized in the trial can be found in Table 1.

Multiple processes will ensure a high level of data quality. Instruments and items were selected by expert researchers, and assistants will be present when participants are filling out the questionnaires, to answer questions and/ or support participants. In addition, data will be quality checked at the research team on a regular basis which includes the checking of adherence to inclusion criteria and checking the completeness and plausibility of data as well associated study documents like the consent form. Data auditing will be administered in form of reviews of the data collection across baseline and three follow-up assessments. The research team will monitor data and document serious adverse events.

The data monitoring committee for the trial (DMC) consists of three well-established, expert researchers that are independent from our funding institution and do not have competing interests: Prof. Dr. Toralf Kirsten (University of Applied Sciences Mittweida), Prof. Dr. Georg Schomerus (Leipzig University), and Prof. Dr. Hans-Helmut Koenig (University Medical Center Hamburg-Eppendorf). The research team will report to the DMC on a regular basis, and the DMC will be involved in the planning, implementation, evaluation, and monitoring of the trial.

We expect to create higher rates of questionnaire returns in the follow-ups by providing assistants that can support participants that have questions regarding the questionnaires.

The collection, storage, and analysis of study data will be carried out in compliance with the relevant data-protection regulations, especially the *DSGVO*. Collected data will be entered in a database using a statistics software package (Stata) and stored locally and password protected. To ensure completeness and accuracy of data entry, a double entry check will be performed. Each participant will receive a pseudonym (project-ID) that will allow us to connect data from different time points and to integrate data from the clinic. The document that connects personal data and project-IDs will be password protected, stored separately from the data, and destroyed at the end of the study. This allows entering and analyzing the collected data in a strictly pseudonymous form. Results will be published in anonymous form, and data will be archived on servers at the University of Leipzig. The research team and the members of the study group at the clinic will have access to the final trial data set.

No interim analyses are planned.

### Adverse events

The participants will be requested to immediately inform their coaches/ hospital staff about serious adverse events, and the occurrence of serious adverse events will be documented. The risk for the occurrence of adverse events through study participation is estimated to be minimal.

### Statistical methods

All data will be examined with regard to potential inconsistencies and missing values. Missing information in variables will be inspected and addressed by using multiple imputation methods [43], if appropriate. In order to check for systematic differences between completers and non-completers we will perform a dropout analysis. In addition, we will check if there are systematic differences between IG and active CG with regard to sociodemographic, health-related and employment variables. Analyses on primary and secondary outcomes will be performed as intention-to-treat. In addition, we will also perform a “per-protocol”-analysis including all participants from the IG that completed the goal agreement and their first intervention and participants from the active CG. Treatment effects on primary and secondary outcomes will be tested using appropriate panel-data regression models including group, time, an interaction between group and time as predictors and adjusting for relevant covariates and baseline outcome measures. Acceptability, uptake and adherence with regard to the intervention will be evaluated using descriptive statistics.

For all analyses the level of statistical significance will be set to *p* < .05. The results of the study will be reported according to the guidelines of *the Consolidated Standards of Reporting Trials* (*CONSORT*) statement [44].

### Dissemination

The study will provide results and materials that will be disseminated nationally and internationally. Study results will be published in peer-reviewed journals and presented at relevant national and international conferences. Additionally, the intervention will be promoted among relevant media. Dissemination activities are led by the researchers at ISAP, and all project partners can contribute to the publication and dissemination of trial results. Authorship will be based on researcher’s contributions.

### Research ethics approval and protocol amendments

The trial will be performed according to the Guidelines for *Good Clinical Practice* (*ICH-GCP*), the *Declaration of Helsinki* and international and local laws. The trial was approved by the ethics committee of the University of Leipzig on 15.12.2020 (531/20-ek).

Any modifications to the protocol which may have an impact on study conduct, intervention design, outcomes, or participant safety will require a formal amendment to the protocol. Major amendments need to be communicated to all parties involved and require the involvement of the ethics committee of the University of Leipzig. Accordingly, the entry at the *German Clinical Trials Register* (DRKS00023245) will be updated. This study protocol is the first version (1.2.2021).

## Discussion

Our study is the first RCT in a German setting where the effects of IPS are tested with a broad group of individuals with severe mental illnesses receiving means tested benefits. We will analyses in how far the treatment affects the integration into the labor market as well as a wide set of vocational and mental health outcomes. The results of this RCT will fill a research gap, but they will also provide the scientific foundation for future measures to improve the reintegration and mental health of long-term unemployed persons with severe mental health problems. The demand for reintegration measures is already existing as a recent German study shows that two thirds of unemployed individuals with a severe mental illness exhibit a strong desire for work [45]. Our trial has several strengths and weaknesses.

One major strength is the fact that we are conducting an RCT, the gold standard in terms of methodology, and that participants are assessed at four points in time. Furthermore, we are assessing a wide variety of outcomes related to employment as well as mental health. This is important since an intervention as complex as IPS may affect a variety of outcomes. Furthermore, there is variation between IPS coachings, e.g. in terms of duration, which could have an effect on outcomes. We address this to some extent by additionally applying per protocol analysis with minimum requirements in terms of intervention attendance, and by rigorously documenting the process.

Another advantage of our study is the collaboration between three distinct actors, clinic, job center, and research institute, that includes project organization and staff training as well as communication and feedback, and a variety of other activities. The lessons learnt from this work can be used to further improve the collaboration in this area and to help create a more efficient and supportive environment for unemployed individuals with mental disorders. However, the COVID19 pandemic may challenge certain procedures and the subsequent labor market.

## Data Availability

After publication of the final results, parts of the datasets will be made accessible in anonymous form to interested researchers upon reasonable request after signing a non-disclosure agreement.

## Abbreviations

CG: Control group
DSGVO: General Data Protection Regulation [German = Datenschutz-Grundverordnung]
IG: Intervention group
IPS: Individual placement and support
LIPSY: Leipzig Individual Placement and Support for people with mental illnesses
RCT: Rrandomized controlled trial
TAU: Treatment as usual
